# Emergency personnel neuroticism, health and lifestyle: A UK Biobank study

**DOI:** 10.1093/occmed/kqz169

**Published:** 2020-01-17

**Authors:** M Mutambudzi, P Flowers, E Demou

**Affiliations:** MRC/CSO Social and Public Health Sciences Unit, Institute of Health and Wellbeing, University of Glasgow, Glasgow, UK

**Keywords:** Chronic disease, emergency personnel, health behaviour, neuroticism, personality, self-rated health

## Abstract

**Background:**

Emergency personnel face unpredictable and challenging incidents and their resilience and ability to cope influences their well-being. Personality traits, such as neuroticism, are postulated to be robust predictors of health and health behaviours. Despite evidence in the general population that neuroticism can positively impact health and health behaviours; to date neuroticism in emergency personnel has primarily been associated with adverse health outcomes.

**Aims:**

To assess whether neuroticism has a negative or positive impact on subjective and objective health and health behaviours in emergency personnel.

**Methods:**

This study used cross-sectional UK Biobank baseline data of emergency personnel (police, firemen and paramedics). Logistic regression models examined the strength of the associations of neuroticism tertiles with subjective (self-reported overall health and chronic conditions) and objective health (abdominal obesity) and self-reported smoking, sleeping, alcohol use and exercise levels.

**Results:**

High neuroticism was positively associated with poorer subjective health outcomes in all emergency personnel (*n* = 2483). The association between neuroticism and chronic disease/s was significant for police in the second (odds ratio [OR] = 1.93, 95% confidence interval [CI] = 1.15–1.94) and third (OR = 1.62, 95% CI = 1.21–2.16) neuroticism tertiles. Neuroticism in firemen was associated with reduced abdominal obesity (OR = 0.49, 95% CI = 0.25–0.96) and increased exercise (OR = 2.14, 95% CI = 1.07–4.25).

**Conclusions:**

We observed positive and negative associations between neuroticism and health outcomes and behaviours. While differences were observed across the emergency personnel groups, more research is needed to better understand how personality traits may impact health in workers with physically and mentally intense jobs.

**Key learning points**

**What is already known about this subject:**

- Emergency personnel are constantly exposed to critical operational and organizational risk factors that can make them susceptible to poor mental health and adverse physical health issues.
- Personality traits, such as neuroticism, are postulated to be robust predictors of health and health behaviours and to influence workers responses to occupational risk factors.
- To our knowledge, to date no studies have assessed the differential impact of neuroticism on health and health behaviours across emergency personnel professional groups.

**What this study adds:**

- This study assesses whether the personality trait of neuroticism has a negative or positive association with subjective and objective health and health behaviours in emergency personnel.
- Overall, high neuroticism was associated with both negative (fair/poor subjective health and chronic diseases) and positive health outcomes (waist circumference) in emergency personnel and negative (police: smoking, sleep deprivation; paramedics: alcohol use) and positive (firemen: physical activity) health behaviours across the different emergency personnel groups.
- High neuroticism was associated with increased reports of poor/fair self-rated health in all emergency personnel, with the strongest associations among firemen.

**What impact this may have on practice or policy:**

- While differences were observed across the emergency personnel groups, more research is needed to determine whether these differences are meaningful and what can be done to improve the health, safety and work ability in demanding professions.

## Introduction

Occupational risks and hazards are widespread and their impact on mental and physical health, well-being and occupational outcomes may be modified by individual differences in personality and genetic liability [1,2]. The work of the emergency services (police, fire, ambulance), compared to other occupations, is compounded by frequent exposure to inherently dangerous, unpredictable and challenging incidents, which require appropriate physical and mental capacities to address effectively [3]. This constant exposure to critical operational and organizational risk factors can make emergency personnel susceptible to poor mental health and adverse physical health issues [4].

Individual coping strategies and processes are developed over working lives and emergency personnel develop a number of traits that allow them to cope effectively (adaptively) with operational and organizational risk factors and attempt to reduce or manage stress within their role [5]; or ineffectively (maladaptively) that can subsequently result in the manifestation of maladaptive behavioural reactions [3]. Personality traits are postulated to be robust predictors of health and health behaviours [1] and are thought to influence workers responses to occupational risk factors in various occupations, including in the emergency services [2,4]. Work-related stress, tension and burnout, coping strategies and job satisfaction, while primarily associated with organizational structures and work environments, have also been associated with personality traits, determining one’s perceptions of and reactions to hazardous situations and ultimately affecting well-being [6]. In particular, neuroticism, defined as the tendency to experience negative thoughts and feelings, insecurity and emotional distress [3,6], has been associated with adverse health outcomes and is reported to impact subjective health more strongly than objective health [7]. Several studies however report that neuroticism may have positive health impacts [1,8,9].

Studies investigating associations between neuroticism and mortality, for instance, vary by populations and outcomes [2,6,10–13]. In the general population, using UK Biobank data neuroticism was positively associated with better mortality outcomes [8,14]. Specifically, higher neuroticism was associated with an 8% reduction in all-cause mortality and reductions in mortality from cancer, cardiovascular and respiratory diseases [8,14]. Higher neuroticism scores related to worry and vulnerability were associated with lower mortality in those reporting fair/poor self-rated health [8]. For health behaviours, general population studies have demonstrated associations between high neuroticism and poor sleep [15], lower engagement in physical activity [16] and with increased risky alcohol use, heavy alcohol consumption and future alcohol problems [17].

Research on neuroticism in emergency personnel, however, has mainly focussed on negative health and work outcomes [2,6,10–13]. A study of British police found neuroticism to be positively related to perceived stress factors, including bureaucracy, politics and interpersonal conflicts; it displayed a direct and positive relationship with emotional coping strategies, including escape and denial; and directly affected well-being and work attitudes [6]. In highway patrol officers [10] and police [12], neuroticism independently predicted and resulted in higher risks of burnout, respectively. Higher neuroticism scores were associated with increased reporting of physical symptoms and emotional exhaustion and negatively correlated with total job satisfaction [10]. When examining depressive symptoms in police recruits 1-year post start of service, greater neuroticism scores at training were associated with greater levels of ‘current’ symptoms of depression and post-traumatic stress disorder (PTSD) and feelings of self-worth [11]. Personality traits have also often been linked to physical symptoms, including cardiovascular reactivity and musculoskeletal disorders [4,13]. A study of operational ambulance personnel demonstrated that neuroticism predicted increased complaints across all studied health outcomes of emotional exhaustion, psychological distress and musculoskeletal pain [4].

It has been proposed that emergency personnel choose their careers because they have certain personality traits that differ from the average person [18] and that personality could be an individual characteristic that influences resilience to occupational risk factors [12]. While studies have examined the impact of personality on health outcomes of emergency personnel, and personnel of other demanding occupations, no studies have assessed the impact of neuroticism on both objective and subjective health and health behaviours in emergency personnel. It is currently unclear how personality traits such as neuroticism impact health and health behaviours among working adults chronically exposed to physically and mentally demanding workloads and unpredictable or dangerous incidents. Further, to our knowledge, no studies assessing the differential impact of neuroticism on health and health behaviours across emergency personnel professional groups have been conducted. To address these knowledge gaps, we (i) investigate whether neuroticism has a negative or positive impact on subjective (i.e. self-rated health) and objective health (self-reported physician-diagnosed chronic diseases and waist circumference) and a range of health behaviours (sleep deprivation, alcohol consumption, smoking status and physical activity) in emergency personnel and (ii) how this may differ across the emergency services professional groups.

## Methods

Baseline UK Biobank data collected from 2006 to 2010 were used for the analysis. The UK Biobank (www.ukbiobank.ac.uk) is a UK-wide, on-going, prospective cohort study established for identifying determinants of disease in middle- and old-age adults [8]. Briefly, over 9 million adults aged 40–69, who lived within 25 miles (40.23 km) from 21 assessment centres and were registered with the National Health Service in England, Wales and Scotland, were asked to participate. Total sample size at baseline was 502 000 representing a 5.5% response rate [19]. Approximately 287 213 were currently employed at baseline, and of these 2483 were emergency personnel (police, paramedics, firemen). Participants were required to visit an assessment centre to complete a computer-assisted self-administered questionnaire, participate in a face-to-face interview and provide physical measures and biological samples.

The UK Biobank study was approved by the North West Multicentre Ethics Research Committee; participants provided written informed consent for data collection and analysis. This research has been conducted using the UK Biobank Resource under Application Number 17333.

The explanatory variable of interest was neuroticism, which was assessed using the 12-item short-form version of the Eysenck Personality Inventory Neuroticism scale—Revised Short Form [8]. This scale has been validated and widely used in working populations and in middle- and old-age adults. The summed score of the 12 questions ranges from 0 to 12 with higher scores indicating higher neuroticism. For the purposes of this study, this was divided into tertiles whose mean neuroticism scores were tertile 1: 0.91 (standard error [SE] = 0.00), tertile 2: 3.95 (SE = 0.003) and tertile 3: 7.97 (SE = 0.01), respectively.

Baseline self-rated health, self-reported physician-diagnosed chronic disease and waist circumference were the health outcomes of interest. To assess self-rated health, participants were asked to rate on a four-point Likert scale their overall health. Responses, coded as ‘excellent’, ‘good’, ‘fair’ or ‘poor’, were dichotomized for this study to ‘excellent or good’ ‘fair or poor’ health. The chronic disease variable indicated the independent or comorbid presence of diabetes, hypertension, heart disease, lung disease and cancer, ascertained through self-report. Waist circumference measurements were taken by trained staff using the Wessex non-stretchable sprung tape measure at baseline [19]. A waist circumference greater than 102 cm for men, and 88 cm for women was classified as abdominally obese, in accordance with WHO classifications. Behavioural outcomes of interest included sleep deprivation (<8 h of sleep), alcohol consumption (consuming alcohol one or more times a week), currently smoking and physical activity performed at least three times a week.

Additional baseline covariates of interest included socio-demographic factors (age, gender, marital status, gross household income), occupational factors (shift work, manual work, work hours, job tenure) and social support (ascertained from responses to the question ‘how often are you able to confide in someone close to you?’). All health behaviours (i.e. sleep deprivation, alcohol consumption, smoking status and physical activity) were included in each of the models assessing health outcomes.

Missing data: Using data for the entire working population at baseline, two approaches were used to account for missing data. First the variable income had approximately 9% missing data, which was socially patterned. To address concerns of non-response bias, and potential systematic underestimation [20], we included participants with missing income observations by creating an income category for missing values. After addressing the missingness of income data, approximately 97% of cases had complete data, and item non-response ranged from approximately 0.01% to 1.5% for each question. To account for the remaining missing data, we imputed missing data in lieu of complete-case analyses, to avoid biased estimates and inflated SEs. Diagnostic tests indicated that data were missing at random. We used multiple imputation by chained equations (MICE) to estimate probable value ranges for incomplete observations. MICE allows for each variable containing missing data to be regressed on all other variables [21] and has been demonstrated to produce asymptotically unbiased estimates and SEs [21]. Five imputed datasets were generated and thereafter assessed according to multiple imputation procedures [22].

Sample characteristics were summarized using frequencies and means. Logistic regression models for which odds ratios (ORs) and 95% confidence intervals (CIs) were reported examined the strength of association between neuroticism and (i) self-rated health, (ii) chronic health conditions and (iii) abdominal obesity. We also estimated models assessing the strength of association between neuroticism and the health behaviours: (i) smoking, (ii) engaging in exercise, (iii) sleep deprivation and (iv) alcohol use. All analyses were performed using Stata14 MP Software (Stata, College Station, TX).

## Results

Overall, our sample was predominantly male (81%), 43% had a gross household income greater or equal to £52 000, 80% lived with a partner and mean age was 48.5 years (SE = 0.12). Approximately 45% of participants were in neuroticism tertile 1, 30.65% in tertile 2 and 24% were in the highest tertile (Table 1). There were no statistically significant differences in mean neuroticism scores of police (mean score = 3.49, SE = 0.07), paramedics (mean score = 3.60, SE = 0.07) and firemen (mean score = 3.40, SE = 0.07).

**Table 1.** Baseline descriptive characteristics

	Neuroticism			
	Tertile 1 %	Tertile 2 %	Tertile 3 %	*P*
Baseline characteristics	44.91	30.65	24.45	
Age (mean and SE)	48.74, 0.18	48.51, 0.22	48.29, 0.25	
Sex				0.00
Female	15.34	19.45	24.05	
Male	84.66	80.55	75.95	
Gross household income				0.11
≤£30 999	10.49	11.70	13.01	
£31 000–51 999	39.73	36.93	42.01	
≥£52 000	45.20	44.94	40.20	
Not reported	4.57	6.44	4.78	
Partner				0.00
Yes	83.32	81.08	76.28	
No	16.68	18.92	23.72	
BMI				0.46
Normal	19.46	18.82	22.24	
Overweight	53.69	53.42	49.92	
Obese	26.85	27.76	27.84	
Alcohol >1 a week				0.58
No	24.22	25.89	26.19	
Yes	75.78	74.11	73.81	
Smoke				0.00
No	49.51	43.42	38.02	
Yes	50.49	56.58	61.98	
Sleep				0.00
7+ h	81.35	73.19	70.35	
≤6 h	18.65	26.81	29.65	
Social support (daily confidant)				0.00
Almost daily	66.39	59.60	44.93	
Once or more a week	14.69	16.78	20.78	
≤Once a month	18.92	23.62	34.29	
Shift				0.28
Never/rarely	22.26	21.08	22.73	
Sometimes	23.07	22.92	26.69	
Usually/always	54.67	55.99	50.58	
Manual work				0.19
No	42.69	46.25	46.46	
Yes	57.31	53.75	53.54	
Weekly work hours (mean and SE)	42.20,0.27	42.16, 0.35	42.16, 0.41	
Tenure in years (mean and SE)	19.30, 0.28	18.95, 0.34	18.65, 0.37	
Chronic health				0.01
No chronic disease	72.91	67.02	66.72	
One or more	27.09	32.98	33.28	
Self-rated health				
Excellent/good	88.25	78.45	67.22	
Fair/poor	11.75	21.55	32.78	
Abdominal obesity				0.302
No	72.50	70.66	69.03	
Yes	27.50	29.34	30.97	
Mean neuroticism score	Mean (SE)	Mean (SE)	Mean (SE)	
Police	0.80 (0.03)	3.93 (0.04)	7.88 (0.08)	
Firemen	0.76 (0.06)	3.97 (0.07)	7.77 (0.16)	
Paramedics	1.00 (0.06)	3.90 (0.08)	7.73 (0.16)	

The logistic regression results examining the association between neuroticism and health outcomes are presented in Table 2. Relative to neuroticism tertile 1, those with higher neuroticism were more likely to report their overall health as being fair/poor. In pooled analyses, participants in the second and third neuroticism tertile were more likely to report fair/poor self-rated health and one or more chronic health condition. When analyses were conducted separately by emergency personnel occupational groups, police officers in tertile 2 and 3 had a 93% (95% CI = 1.38–2.71) and an almost 3-fold (95% CI = 2.08–4.16) increased likelihood of reporting fair/poor health. Among paramedics, neuroticism tertile 3 was associated with poorer self-reported overall health (OR = 4.63; 95% CI = 2.28–9.40). Firemen indicated a gradient effect of increasing adverse health when each tertile threshold was crossed. Firemen in tertile 2 (OR = 3.44; 95% CI = 1.77–6.68) and 3 (OR = 5.53; 95% CI = 2.77–11.04) were most likely to self-report fair/poor overall health. The association between neuroticism and risk of one or more chronic disease/s was significant for police only (Table 2). High neuroticism was protective of abdominal obesity in firemen in the third tertile only (OR = 0.49; 95% CI = 0.25–0.96).

**Table 2.** Association between neuroticism and health outcomes in emergency personnel^a,b^

	Pooled data			Police officers *n* = 1642			Paramedics *n* = 360			Firemen *n* = 481		
	OR	95% CI		OR	95% CI		OR	95% CI		OR	95% CI	
Poor/fair self-rated health												
Neuroticism												
2	2.03	1.55	2.67	1.93	1.38	2.71	1.52	0.73	3.19	3.44	1.77	6.68
3	3.37	2.56	4.44	2.95	2.08	4.16	4.63	2.28	9.40	5.53	2.77	11.04
Chronic health conditions (one or more)												
Neuroticism												
2	1.40	1.13	1.73	1.49	1.15	1.94	1.39	0.78	2.46	1.14	0.67	1.95
3	1.42	1.12	1.79	1.62	1.21	2.16	1.44	0.79	2.61	0.92	0.50	1.67
Abdominal obesity												
Neuroticism												
2	0.94	0.76	1.17	0.91	0.70	1.19	1.35	0.75	2.41	0.86	0.48	1.52
3	0.84	0.66	1.08	1.00	0.74	1.34	0.67	0.34	1.30	0.49	0.25	0.96

^a^Neuroticism reference category was tertile 1.

^b^Fully adjusted for all covariates and health behaviours.

Table 3 presents the results of the association between neuroticism and health behaviours. In pooled analyses, participants in the second and third tertile of neuroticism exhibited a 28% (95% CI = 1.05–1.56) and 56% (95% CI = 1.25–1.94) increased risk of smoking, respectively. Stratified analyses indicated that only police in tertile 3 had a statistically significant increased risk of smoking (OR = 1.77; 95% CI = 1.35–2.32), while high neuroticism (tertile 3) was associated with increased alcohol consumption in paramedics only (OR = 2.27; 95% CI = 1.15–4.48). Further, sleep deprivation with increasing neuroticism was evident in police officers (OR = 1.45; 95% CI = 1.09–1.92), while high neuroticism was associated with more exercise in firemen only (OR = 2.14; 95% CI = 1.07–4.25).

**Table 3.** Association between neuroticism and health behaviours in emergency personnel^a,b^

	Pooled data			Police officers *n* = 1642			Paramedics *n* = 360			Firemen *n* = 481		
	OR	95% CI		OR	95% CI		OR	95% CI		OR	95% CI	
Smoking												
Neuroticism												
**2**	1.28	1.05	1.56	1.27	1.00	1.60	0.99	0.56	1.75	1.55	0.98	2.45
3	1.56	1.25	1.94	1.77	1.35	2.32	0.90	0.49	1.65	1.36	0.80	2.28
Exercise												
Neuroticism												
**2**	1.15	0.93	1.43	1.09	0.85	1.41	1.19	0.61	2.31	1.47	0.77	2.80
3	1.37	1.08	1.74	1.22	0.92	1.62	1.84	0.92	3.66	2.14	1.07	4.25
Sleep deprivation												
Neuroticism												
2	1.47	1.17	1.86	1.45	1.09	1.92	1.59	0.81	3.14	1.60	0.92	2.80
3	1.68	1.31	2.16	1.81	1.34	2.45	1.78	0.86	3.67	1.50	0.80	2.80
Alcohol consumption												
Neuroticism												
2	1.02	0.82	1.28	0.90	0.68	1.18	1.06	0.59	1.91	1.58	0.89	2.82
3	1.07	0.83	1.38	0.90	0.66	1.22	2.27	1.15	4.48	1.23	0.66	2.28

^a^Neuroticism reference category was tertile 1.

^b^Fully adjusted for all covariates and health behaviours.

## Discussion

Our study, for the first time to our knowledge, assesses whether the personality trait of neuroticism has a negative or positive impact on subjective and objective health and health behaviours in emergency personnel. Overall, high neuroticism was associated with both negative (fair/poor subjective health and chronic diseases) and positive health outcomes (waist circumference) and negative (police: smoking and sleep deprivation; paramedics: alcohol use) and positive behaviours (firemen: physical activity). High neuroticism was associated with increased reports of poor/fair self-rated health in all emergency personnel, with the strongest associations among firemen. Examination of neuroticism’s impact on the objective health measure of abdominal obesity, however, showed a decreasing risk of adverse outcomes with high neuroticism in firemen, and no significant association in other personnel. Previous research has reported similar outcomes and indicated stronger relationships between neuroticism and perceptions of health status, than actual health, and therefore affects subjective health more strongly than objective health [7,23]. According to the literature, individuals with high neuroticism may be more vigilant, internally focussed and pain-sensitive [23], resulting in heightened symptom perception which may lead to attentional bias [7]. Firemen with high neuroticism reported a 5-fold increased risk in self-reported poor health compared to those with low neuroticism. Despite being healthier and engaging in more physical activity, we postulate that they may overestimate their susceptibility to disease due to chronic exposure to pollutants.

While the role of chance in shaping these findings cannot be ruled out, our data suggest that there are differences between the emergency personnel groups. We observed a greater risk of negative health outcomes and behaviours among police and paramedics in higher neuroticism tertiles, and more positive objective health outcomes and health behaviours among firemen. The reasons for this finding are unclear, and more research would be needed to determine if it is meaningful. For instance, these differences could possibly reflect differences in workplace settings and work organization, rather than and/or in addition to personality trait differences.

Emergency service personnel do physically and mentally demanding work, characterized by irregular and often dangerous incidents [6,24], and it is associated with stress, fatigue and burnout, factors which increase the susceptibility of poor health and risky health behaviours. Firemen need to be in good physical health and have a high level of physical fitness to carry out their duties. During a work shift, occupational demands are generally low, with episodes of intense demands that require high energy and fitness levels [24]. The physically demanding tasks firemen engage in, all while wearing heavy and bulky personal protective equipment [24], may contribute to a decreased risk of adiposity. In addition, given the high job demands, coupled with long periods of low occupational activity, it is possible that fire departments may provide more opportunities for voluntary exercise during the workday. Further, the demanding nature of the job activities may also encourage those with high neuroticism, who are more prone to being vigilant and/or channel their anxiety into positive health behaviours, to be proactive with their physical fitness.

Some studies have reported lower levels of stress and burnout in firemen relative to police officers, which may in part be attributed to work arrangements and organizational resources and structure [25,26]. While both professions engage in shift work, fire department shifts are often 24-h long, followed by 48-h rest periods, while police department shifts often have fast rotations without guarantee of long rest periods between [26,27]. Firemen therefore may have sufficient time to mentally and physically recuperate and engage in health-promoting behaviours. In addition, this shift pattern in the fire service may provide unique opportunities for colleagues to develop strong bonds and high work cohesion. This social support is not only significantly associated with decreased anxiety and greater effectiveness in responding to emergencies but also increased psychological and physical well-being [25,28]. We found a greater risk of alcohol consumption in paramedics, however, caution should be taken in interpreting this finding as it may be tempered by the relatively smaller number of paramedics, potentially diminishing statistical power. We are therefore unable to offer a steadfast conclusion regarding this finding and recommend that future studies include a larger sample of paramedics.

Our findings should be considered in the light of several limitations. Use of self-reported chronic health conditions has often been criticized as it can be influenced by disease awareness and recall bias among other factors. Previous research however has found self-reported doctor-diagnosed outcomes to be highly correlated with medical records [29]. In addition, factors known to negatively affect health (e.g. work flexibility, supportive work environment and age, gender, racial discrimination at work) were not available. A further limitation is the use of cross-sectional data, which limits our ability to assess any cumulative effects. Finally, we could not account for other personal and organizational factors that can moderate the relationship between personality and health. Therefore, despite having similar personality traits, there may be individual differences in responses and attitudes towards risk factors or organizational and setting-specific factors, which when not accounted for reduce the precision of estimated effects on health outcomes and behaviours [28].

Despite these limitations, our findings are strengthened using a large, national, cohort with relatively large sample size. Personality traits are postulated to be robust predictors of health and health behaviours and may provide more insight into susceptibility to adverse health, and the barriers or facilitators of health-promoting behaviours in different groups and occupational settings [1]. More research is needed to better understand how personality traits may impact health in workers with physically and mentally intense jobs.

The results of this study highlight the importance of including personality in occupational and public health research. Previous research suggests a significant direct relationship between personality traits and the type of coping strategies police officers use [6]. Neuroticism in particular is associated with an increase in reactivity to threats, burnout and stress in the workplace. Understanding the association between personality and health in workers with physically and mentally intense jobs and how the workplace setting can impact on this complex relationship may provide opportunities for workplace interventions. In relation to making recommendations for future interventions, the results presented here should not be taken to translate straightforwardly into any particular single intervention approach. Instead these results may be useful as one contributing element in the future co-production of interventions that balance evidence of effectiveness, evidence of causal mechanisms underpinning health outcomes (e.g. neuroticism) with wider issues of intervention implementation and sustainability. For example, approaches such as psychoeducation with individuals, wider teams and the whole organization about neuroticism and its implications for health and well-being could be combined with the provision of mindfulness-based stress reduction programs that strengthen personal outlook, encourage resilience, optimism and hope and promote effective coping strategies, as these have been found to be effective and can be applied in a wide range of settings including workplaces [30].

## Funding

We acknowledge financial support from the Medical Research Council and Chief Scientist Office (MC_UU_12017/12; SPHSU12) and an MRC Strategic Award (MC_PC_13027 for E.D.).

## Competing interests

E.D. has previously received part funding to conduct a different research project on police officer well-being.
